# New traits in crops produced by genome editing techniques based on deletions

**DOI:** 10.1007/s11816-017-0425-z

**Published:** 2017-02-13

**Authors:** C. C. M. van de Wiel, J. G. Schaart, L. A. P. Lotz, M. J. M. Smulders

**Affiliations:** Wageningen University and Research, Wageningen, The Netherlands

**Keywords:** Nuclease, SDN, SSN, Precision breeding, Sustainable agriculture

## Abstract

One of the most promising New Plant Breeding Techniques is genome editing (also called gene editing) with the help of a programmable site-directed nuclease (SDN). In this review, we focus on SDN-1, which is the generation of small deletions or insertions (indels) at a precisely defined location in the genome with zinc finger nucleases (ZFN), TALENs, or CRISPR-Cas9. The programmable nuclease is used to induce a double-strand break in the DNA, while the repair is left to the plant cell itself, and mistakes are introduced, while the cell is repairing the double-strand break using the relatively error-prone NHEJ pathway. From a biological point of view, it could be considered as a form of targeted mutagenesis. We first discuss improvements and new technical variants for SDN-1, in particular employing CRISPR-Cas, and subsequently explore the effectiveness of targeted deletions that eliminate the function of a gene, as an approach to generate novel traits useful for improving agricultural sustainability, including disease resistances. We compare them with examples of deletions that resulted in novel functionality as known from crop domestication and classical mutation breeding (both using radiation and chemical mutagens). Finally, we touch upon regulatory and access and benefit sharing issues regarding the plants produced.

## Introduction

New plant breeding techniques (NPBT) encompass a set of diverse techniques and concepts that all aim to improve efficiency and/or precision of plant breeding. Most make use of transgenic plant lines at some point in the breeding process, but the final product generally contains only small mutations and in specific cased no modifications at all, and is often indistinguishable from the conventional breeding products (Lusser et al. 2012; Schaart et al. 2016). One of the most promising among these, genome editing (also called gene editing or gene targeting) with the help of a programmable nuclease (SDN: Site-directed nuclease, or SSN: sequence-specific nuclease), recently has led to a deluge of creative applications with the introduction of the CRISPR-Cas9 system. While the thorough review of NPBT by Lusser et al. (2012) not yet mentioned CRISPR-Cas, 4 years later a search using CRISPR and plants as keywords provided 246 publications in Web of Science (on 31-10-2016). Similarly to zinc finger nucleases (ZFN) and TALENs, CRISPR-Cas is able to make a double-strand break (DSB) at a precisely specified location in the genome, but it is much more versatile and easy to use, because the specificity of the target sequence is achieved by a separate guide RNA (gRNA) that can be easily designed and readily synthesised rather than by the protein structure itself (ZFN, TALEN). The use in plants has recently been reviewed by Luo et al. (2016), Paul and Qi (2016), Hilscher et al. (2016), and Rani et al. (2016).

At an early stage of the development of genome editing, three types of uses based on the DSB repair mechanism have been distinguished for regulatory purposes (Lusser et al. 2011). In SDN-1, the DSB is repaired by the non-homologous end joining (NHEJ) DNA repair machinery of the cell, during which small mistakes may be introduced, mostly consisting of small indels. Plants with such mistakes can be identified by screening. Indels in genes frequently lead to gene knockout as a result of reading frame shifts causing premature translational stops. In SDN-2, an oligonucleotide is provided to assist in the repair of the DSB that is identical to the sequence in which the DSB is made, except that a desired mutation is included. Some plants may use this oligo in an alternative cellular repair mechanism, homology-directed repair (HDR). This may lead to a higher frequency of plants with the desired change, e.g., a single amino-acid substitution. In SDN-3, the DSB is repaired in the same way, but the repair template is a longer sequence that may include a complete gene or a promoter. This will lead to the insertion of native or foreign sequences at a precisely specified location in the genome. SDN-3 is thus similar to genetic transformation and it generally results in transgenic plants, but they are produced with improved precision as the inserted sequence is targeted to a specific site in the genome, or exchanged with existing sequences at that site, e.g., in promoter swaps. Thus, SDN-3 holds much promise for diverse applications, including stacking desirable (transgenic) traits, so that they are passed on to progeny as a single block during breeding, which is desirable for efficient introgression into diverse elite materials (Kumar et al. 2016). HDR is still a relatively difficult to control mechanism. An alternative technique is called ODM (oligo-directed mutagenesis). It may produce results similar to SDN-2 but does not employ a nuclease to make a DSB. Combinations of both, improving efficiency, are also explored (Sauer et al. 2016).

Although SDN-1 is precise only in the targeted site and not in the resulting type of mutation, it appears presently to be the most frequently implemented, and is, therefore, the subject of this review. We first discuss improvements and new variants of SDN-1, in particular employing CRISPR-Cas, and subsequently explore the possibilities of targeted deletions that eliminate the function or part of the function of a gene, as an approach to generate novel traits useful for improvement of agricultural sustainability. We compare them with examples of deletions that resulted in novel functionality as known from crop domestication and classical mutation breeding. Finally, we touch upon regulatory and IP issues around the plants produced, as well as access and benefit sharing.

## Improvements and new variants of the technology

### Off-target effects

CRISPR-Cas genome editing has been reported to be accompanied by off-target effects, i.e., mutations arising from repairing DSBs induced elsewhere in the genome than the targeted sequence. Several research groups have made changes to the Cas protein and/or gRNA design to enhance the sequence target specificity (Kleinstiver et al. 2016; Slaymaker et al. 2016; Doench et al. 2016). In addition, alternative endonucleases (e.g. Cpf1) could increase specificity (e.g., Ran et al. 2015; Kim et al. 2016a). Schiml et al. (2014) turned Cas9 into a nickase, so that two adjacent gRNAs were required to make a DSB with sticky ends. This improved the specificity, alone and in combination with using truncated gRNAs (Fu et al. 2014). Tolerance for mismatches in the gRNA (actually off-target effects) may also be used to specifically target related genes from gene families (Endo et al. 2015).

Various methods for genome-wide detection of off-target mutations have been developed, including BLESS (Crosetto et al. 2013), GUIDE-seq (Tsai et al. 2015), Digenome-seq (Kim et al. 2015, 2016b), and END-seq (Canela et al. 2016). For the few plant species assessed so far, little off-target effects appear to occur using CRISPR-Cas, but this would need to be studied in more species (Peterson et al. 2016). For example, off-target effects could be limited using an optimal molar ratio of Cas9 to gRNAs by Woo et al. (2015), while Peterson et al. (2016) did not detect any in a multiplex approach by re-sequencing in *Arabidopsis*, including in computationally predicted sites.

Off-target mutations are considered a problem in, e.g., applications in humans, but they are less likely to pose a problem in plant breeding, as usually multiple plants are produced that are subsequently screened and selected. In many cases, there are multiple generations between the genome editing step and the variety produced, in which off-target mutations are selected against and in seed-propagated crops, segregate out. In addition, the frequency of off-target mutations made across the genome will be much lower than in classical mutagenesis. The type and frequency of mutations in classical mutagenesis depend on the method (chemical or ionizing radiation) used, type and concentration of chemical or type of radiation, and the duration of the treatment, which are generally adjusted case by case (Suprasanna et al. 2015). Polyploid crops, in general, tolerate a higher frequency than diploid crops (Uauy et al. 2009; Shu et al. 2011; Oladosu et al. 2016). A frequency of one mutation induced every 78 kb (what Tsuda et al. 2015 used in soybean) would in soybean or tomato mean that classical mutagenesis may introduce for every desired mutation more than 10,000 other mutations.

### Delivery

Commonly plants are transformed using *Agrobacterium* or biolistic systems to introduce the Cas9- and gRNAs-encoding DNA stably into the plant genome. This may work best in tissue culture systems, as exemplified by the high success rate in rice (Paul and Qi 2016). When using floral dip, germline editing was shown to be improved in *Arabidopsis* by driving the expression of Cas9 with promoters specific for egg cells (Wang et al. 2015) or for actively dividing tissues, such as meristems and embryo sacs (Hyun et al. 2015; Yan et al. 2015). Alternatively, viral vectors for gRNA (Ali et al. 2015; Yin et al. 2015) may improve efficiency.

Clasen et al. (2016) used a temporary expression system, i.e., a TALEN transcribed from plasmids introduced into potato protoplasts. Regenerated plants with mutations were checked for the absence of plasmid sequence insertions in their genome. Woo et al. (2015) transfected protoplasts of *Arabidopsis thaliana*, tobacco, lettuce, and rice with ribonucleoprotein complexes containing Cas9 nuclease protein and appropriate gRNAs and obtained genome-edited regenerated plants. They called their approach “DNA free genome editing”. It has also been used in the unicellular green alga *Chlamydomonas reinhardtii* (Baek et al. 2016) and it was also shown to work in *Petunia hybrida* protoplasts (Subburaj et al. 2016). The approach requires protocols for protoplasting and plant regeneration from protoplasts, which are not available for many crops, or, if existing, not for all varieties within a crop. The desire to use genome editing in research and in plant breeding urges for a surge of activity into transformation, protoplasting, and regeneration protocols for crops and model species that up to now have remained recalcitrant, such as wheat (Altpeter et al. 2016).

### Multiplexing

CRISPR-Cas9 is particularly suitable to multiplexing because of the versatility of the gRNAs, but also TALENs have been multiplexed: four *Nicotiana benthamiana* genes involved in glycosylation were knocked out simultaneously using TALENs for the benefit of biopharmaceutical production of glycoproteins devoid of plant-specific residues (Li et al. 2016). Peterson et al. (2016) targeted 14 loci simultaneously in *A. thaliana* with CRISPR-Cas using stacked gRNA expression arrays (Peterson et al. 2016). Steinert et al. (2015) simultaneously used two alternative modified Cas proteins, and each was directed by its own gRNA.

### Larger deletions

Using two gRNAs targeted to sites at some distance from each other on the same chromosome, larger deletions become possible based on annealing the distant sites through NHEJ. In this way, Zhou et al. (2014) managed to delete a cluster of up to 10 loci in the terpenoid synthesis pathway in rice. Deleting clusters of related genes is useful, e.g., for the removal of alpha- or gamma-gliadins, which are organized in gene repeats on different chromosomes, while trying to produce wheat that is safe for people with celiac disease (Jouanin et al., in preparation).

## Generation of new traits for crop improvement through deletions

‘Classical’ genetic modification was developed to introduce genes into plants to obtain a gain of function, although it soon was also used to silence endogenous genes, e.g., using RNAi. The reason for this is that the loss of a gene or gene function may also result in a new plant phenotype that is useful for man. During crop domestication, many traits have been selected for that are inherited as recessive, so mostly loss-of-function (Lu et al. 2006; Hancock 2012; Martínez-Ainsworth and Tenaillon 2016). They often comprised knockouts of genes. Examples include loss of seed shattering, loss of seed dormancy, reduction of shoots improving the harvest index, reduction of chemical and physical defences, and loss of photoperiodicity and/or the vernalisation requirement (Nakamichi 2015). Similar mutations have been selected for independently in different crops (e.g., Cheng et al. 2016).

Since the 1930s, (knockout) mutagenesis has been used to generate new traits, boosted by increased knowledge on the effects of various sources of radiation (which mostly induce deletions) and chemical mutagens (for example, EMS induces C > T mutations). This had led to the development of more than 3000 crop varieties, and it is still popular, for instance, for generating new flower colours in ornamentals (it is a standard procedure in Chrysanthemum). Interestingly, mutation breeding also produces, at useable frequencies, gain of function traits, such as new disease resistances (Oladosu et al. 2015). Resistance to biotic and abiotic stresses is an important goal of breeding new varieties, as one of the elements to feed the world in a sustainable way.

The SDN-1 systems make it also easier to deal with the recessive nature of the mutations, which in classical mutagenesis generally means that mutations do not show a phenotype in the mutants themselves, only in their progeny. SDN-1 can mutate all alleles of the targeted locus simultaneously, which is a particularly significant improvement in mutating polyploids. Examples are deletions in the three homoeologues (six alleles) of *MLO* for powdery mildew resistance in hexaploid wheat using TALENs by Wang et al. (2014) and the four copies of GBSS for altering starch composition in tetraploid potato by Andersson et al. (2016).

### Disease resistance

Increasing pathogen resistance in crops is an important way to improve agricultural sustainability. Resistance may be generated by deleting plant genes that are used (‘hijacked’) by pathogens and on which the pathogens depend for growth and development (Pavan et al. 2010). These are called S genes (for Susceptibility, in contrast to dominant R genes which confer Resistance through recognition of the pathogen). The classical example is *mlo*, a recessive mutant which has been effective for mildew resistance in barley for over 30 years (reviewed by Acevedo-Garcia et al. 2014).

Knocking out S genes generated disease resistance in multiple crop species, but this may be accompanied by poor plant performance. Precisely targeted deletions using SDN-1 can make it possible to generate specific pathogen resistance without fitness costs for the plant. Using TALENs, Li et al. (2012) were able to identify and delete a small part of the rice SWEET14 promoter that was targeted by an effector protein secreted by the causal agent of Bacterial Leaf Blight, *Xanthomonas oryzae* pv. oryzae (Xoo), apparently without affecting adequate expression of the gene by the plant itself. Thus, a rice line was generated that was resistant to Xoo strains that use this effector. Later on, also CRISPR-Cas9 was implemented for mutating SWEET-type S genes (Zhou et al. 2014). S genes have been identified in a range of species, including *Arabidopsis*, rice, soybean, and potato and tomato (Sun et al. 2016a, 2016b; Zheng et al. 2016).

Resistance to viruses can also be generated following an S gene approach. Disrupting the functionality of eIF4E (eukaryotic translation initiation factor 4E) effected broad potyvirus resistance in cucumber (Chandrasekaran et al. 2016). Pyott et al. (2016) likewise achieved potyvirus resistance in *Arabidopsis*.

### Product quality

In many crops, including legumes and cereals, phytate in seeds interferes with phosphorous uptake. Decreasing phytate content in feed will increase net uptake of P by livestock which may reduce losses of P to the environment. Liang et al. (2014) used both TALEN and CRISPR-Cas9 successfully in maize to obtain mutations in *IPK*, which is involved in phytate production. Reducing phytate comes with trade-offs for plant performance that may be addressed more precisely through genome editing. Recently, Yamaji et al. (2017) reported an alternative approach of reducing seed P content in rice using retrotransposon-insertional knockout mutants of *SPDT*, a P distribution transporter controlling the allocation of P to the grains. In these mutant plants, yield and seed performance were apparently not affected. An alternative TALEN-based approach was explored by Wendt et al. (2013): introducing small indels into the promoter of the most important barley grain phytase gene, *HvPAPhy_a*, which could be used to test for the possibilities of changing gene expression in the grain. Mutations were achieved in the targeted promoter site, but no further testing of plants was reported.

Clasen et al. (2016) used gene editing to knock out *Vnvl* to reduce acrylamide (a potential carcinogen) levels of potato products after heating. A similar result could be obtained by silencing asparagine synthetase genes, in this case using RNAi (Zhu et al. 2016). Some other examples of traits improving product quality targeted by SDN-1 are high amylopectin (“waxy”) maize (by Pioneer, https://www.aphis.usda.gov/biotechnology/downloads/reg_loi/15-352-01_air_response_signed.pdf) and potato (Andersson et al. 2016) based on knockout of *GBSS*, and high oleic acid oilseed crops soybean (Haun et al. 2014) and camelina (Jiang et al. 2016) based on knockouts of *FAD2* and/or *FAD3*. Both traits had been addressed earlier using classical mutagenesis or RNAi. Rice fragrancy was improved by knockout of *BADH2* (Shan et al. 2015). For bio-based crops, reducing lignin contents has been studied using a transgenic silencing method up to the level of field trials in poplar for improving biofuel production (Van Acker et al. 2014). Zhou et al. (2015) reported successful SDN-1 in two poplar 4CL genes, the knockout of one of which led to lower lignin levels in stem wood.

### Allergens

Removing allergens through genome editing would benefit specific groups of consumers. Dubois et al. (2015) showed that silencing Mal d 1 reduced the allergenicity of apple, which, in most patients, is a mild allergy resulting from cross-reactivity of the birch pollen Bet v 1 allergens to PR-10 proteins in Rosaceous fruits, such as apple, cherry, and strawberry. PR-10 proteins are encoded by a large gene family. Apple has 31 Mal d 1 genes of which 20 may be expressed in the fruit (Pagliarani et al. 2013) and may be targeted by genome editing. Peanut allergy is a life-threatening food allergy. Dodo et al. (2008) managed to reduce the allergenicity of the immunodominant Ara h 2 protein in peanuts using RNAi. For hypoallergenic peanuts to be safe for consumption by many patients, all genes coding for allergens would need to be silenced or removed, and genome editing offers the tools to efficiently do this.

Sensitivity of individuals with coeliac disease to cereal gluten is particularly difficult to address as gluten comprises of large gene families, with several epitopes in mainly alpha-, gamma-, and omega-gliadins that can be recognised by human T cells (Van Herpen et al. 2006; Van den Broeck et al. 2009; Salentijn et al. 2012) and that all are targets for controlled deletion or modification (Smulders et al. 2015; Jouannin et al. in prep.). Barro et al. (2016) succeeded in removing highly immunogenic gliadin proteins from wheat using RNAi. Clinical trials using breads from these silenced wheat plants were planned in Spain. Silencing will in most cases not completely remove the gliadin, and a complete knockout approach by genome editing may be more effective, and possibly more easily accepted by consumers as no foreign DNA is introduced (Laursen 2016).

### Hybrid varieties

Pivotal to successful production of high-quality hybrid varieties is the availability of reliably male-sterile maternal lines. Pioneer is using a male sterility system in maize involving mutants of the male fertility gene *Ms45*. Knockout of the *Ms45* gene using CRISPR-Cas9 was recently reported by Svitashev et al. (2015).

### Yield

Interestingly, a complex trait par excellence, yield, was also shown to be amenable to an SDN-1 approach. Li et al. (2017) used CRISPR-Cas9 in rice to mutate the regulatory genes *Gna1, DEP1*, and *GS3* and obtained plants with increased grain numbers, dense erect panicles plus semi-dwarf phenotype, and larger grains, respectively.

## Regulatory and IP issues

Currently, there is worldwide a discussion whether genome-edited plants should fall under existing regulatory systems that have been designed for transgenic (GM or GE) plants (for an overview, see Sprink et al. 2016). In the US, no regulatory oversight was deemed necessary by USDA-APHIS (e.g., waxy maize, high oleic acid soybean mentioned above under ‘Product quality’ and *sweet14*-based blight resistance in rice under ‘Disease resistance’, see for further examples Table 1 of Hilscher et al. 2016). In Europe, there is no definitive legal analysis yet. The EFSA GMO unit (2015) considered SDN-1 a form of mutagenesis; further analysis may be needed upon technological advancement. Among EU Member States, the German BVL (Federal Office of Consumer Protection and Food Safety) had a similar judgement, including for CRISPR, as gRNAs were not seen as recombinant DNA (i.e., no novel combination of genetic material) and the Swedish Board of Agriculture (SBA) saw CRISPR-Cas as equivalent to mutagenesis provided that no “foreign” DNA was left in plants. The German BfN (Federal Agency for Nature Conservation) held a different view, as were NGOs and organic farming organizations (see IFOAM 2015 statement): they consider the process as most relevant and the process involves molecules not occurring naturally (Sprink et al. 2016). Mutagenesis was exempted from regulation in EU Directive 2001/18/EC as having a history of safe use (see for detailed comparison with SDN-1, the off-targets section above).

The EU GM regulation is perceived as prohibitive to most applications and to small companies because of costs and uncertainties around timing and outcomes of the application procedure. When extensive GM regulation would be applicable to SDN-1 techniques, this may lead to a paradoxical result. Parties aiming at commercializing the plant products could produce a gene-edited plant with deletions to establish whether it generates the desired functionality, and then would “re-produce” a similar plant using classical mutagenesis. Companies would be able to do that also because the quickly increasing efficiency of next-generation DNA sequencing for mutation screening in recent times has stimulated them to (re)develop mutated populations in their crops (Van de Wiel et al. 2016). However, this would not be an efficient and optimal use of resources in breeding, and would give up on advantages hardly feasible by classical mutagenesis, such as the possibility of inducing recessive mutations in all alleles of a targeted gene in polyploid crops. There is a need for regulatory systems duly addressing biosafety that are balanced in terms of workability and costs, so that they do not unduly limit the benefits of producing innovative plant products with potential advantages to agricultural sustainability.

The possibilities offered by genome editing also have ramifications to IP, access, and benefit issues, even when it concerns deletions and loss-of-function mutations. It may also raise questions in the context of the use of genetic resources: can copying a mutation already existing in a genebank accession into elite material be considered use in the sense of the ‘*Nagoya Protocol on Access to Genetic Resources and the Fair and Equitable Sharing of Benefits Arising from their Utilization to the Convention on Biological Diversity*’? These ramifications may attract a lot of attention in the coming years when the possibilities of SDN-1 are realized in the form of improved crop varieties (or even varieties from underutilized or novel crops). These discussions are important for the realisation of the possibilities that SDN-1 using genome editing offers for increasing the sustainability of agriculture.
