# Correction to: New traits in crops produced by genome editing techniques based on deletions

**DOI:** 10.1007/s11816-018-0498-3

**Published:** 2018-09-27

**Authors:** C. C. M. van de Wiel, J. G. Schaart, L. A. P. Lotz, M. J. M. Smulders

**Affiliations:** Wageningen University and Research, Wageningen, The Netherlands

## Correction to: Plant Biotechnol Rep (2017) 11:1–8 10.1007/s11816-017-0425-z

In the original publication of the article, Li et al. has been incorrectly cited in the following sentence “Interestingly, a complex trait par excellence, yield, was also shown to be amenable to an SDN-1 approach. Li et al. (2017) used CRISPR-Cas9 in rice to mutate the regulatory genes *Gna1, DEP1*, and *GS3* and obtained plants with increased grain numbers, dense erect panicles plus semi-dwarf phenotype, and larger grains, respectively.”

The sentence with the correct citation is provided.

Interestingly, a complex trait par excellence, yield, was also shown to be amenable to an SDN-1 approach. Li et al. (2016) used CRISPR-Cas9 in rice to mutate the regulatory genes *Gna1, DEP1*, and *GS3* and obtained plants with increased grain numbers, dense erect panicles plus semi-dwarf phenotype, and larger grains, respectively.

The reference is,

Li M, Li X, Zhou Z, Wu P, Fang M, Pan X, Lin Q, Luo W, Wu G, Li H (2016) Reassessment of the four yield-related genes Gn1a, DEP1, GS3, and IPA1 in rice using a CRISPR/cas9 system. Frontiers in Plant Science 7:377. 10.3389/fpls.2016.00377.

